# Diagnosing and Ranking Retinopathy Disease Level Using Diabetic Fundus Image Recuperation Approach

**DOI:** 10.1155/2015/534045

**Published:** 2015-04-07

**Authors:** K. Somasundaram, P. Alli Rajendran

**Affiliations:** ^1^Department of Computer Science and Engineering, PSNA College of Engineering and Technology, Dindigul 624 622, India; ^2^Department of Computer Science and Engineering, Velammal College of Engineering and Technology, Madurai, Tamil Nadu 625 009, India

## Abstract

Retinal fundus images are widely used in diagnosing different types of eye diseases. The existing methods such as Feature Based Macular Edema Detection (FMED) and Optimally Adjusted Morphological Operator (OAMO) effectively detected the presence of exudation in fundus images and identified the true positive ratio of exudates detection, respectively. These mechanically detected exudates did not include more detailed feature selection technique to the system for detection of diabetic retinopathy. To categorize the exudates, Diabetic Fundus Image Recuperation (DFIR) method based on sliding window approach is developed in this work to select the features of optic cup in digital retinal fundus images. The DFIR feature selection uses collection of sliding windows with varying range to obtain the features based on the histogram value using Group Sparsity Nonoverlapping Function. Using support vector model in the second phase, the DFIR method based on Spiral Basis Function effectively ranks the diabetic retinopathy disease level. The ranking of disease level on each candidate set provides a much promising result for developing practically automated and assisted diabetic retinopathy diagnosis system. Experimental work on digital fundus images using the DFIR method performs research on the factors such as sensitivity, ranking efficiency, and feature selection time.

## 1. Introduction

The identification of exudates in the macular region plays a significant part in diabetic macular edema and helps in the effective detection of the severity of the disease with higher level of sensitivity. As a result, exudates detection plays an important task in the efficient diagnosing. In the design of computer based diagnosis and ranking of diabetic retinopathy, many algorithms and techniques for efficient exudates detection were presented and discussed.

The first part of the introductory section provides a detailed discussion of different ways in which the contribution of image processing for efficient diagnosing of diabetic retinopathy has been made by different researchers. In [1], a twofold method for early detection of macular edema was presented with the help of mathematical morphology to identify the fovea region. With the application of mathematical morphology, significant minimization of load was achieved by efficiently capturing the global characteristics of images.

One of the most fundamental reasons for the cause of blindness in the modern world is diabetic retinopathy (DR), of which diabetic macular edema can be processed by efficient detection of exudates present in the fundus images. Feature Based Macular Edema Detection (FMED) [2] introduced a new technique for the effective diagnosing of the DME with the help of novel features, namely, color, decomposition of wavelet, segmentation of lesion, and so on. Even though the technique was proved to be efficient in terms of computation, an efficient feature selection system for transparent disease diagnose was not possible and was not categorized into true positive and true negative ratio.

Macular edema as one of the causes of retina results in the minimization of vision. In [3], a twofold technique for measuring the severity of DME was presented using the classification process for digital fundus images. A supervised learning model was framed using normal digital fundus images, where the extraction of features was performed to obtain the dynamic form of digital fundus images and efficient means were introduced to differentiate between the normal and DME images. Though sensitivity and specificity were improved, the work follows with an assumption that an image is said to be normal if it has no lesions.

In order to effectively measure the severity of retinal diseases, extraction of vessel is also considered as an important means for digital fundus images. Contrast-Limited Adaptive Histogram Equalization (CLAHE) [4] presented a novel method that efficiently segmented the retinal blood vessels that differentiated between the Proliferative Diabetic Retinopathy (PDR) and digital fundus images using 2D-Match (Gabor) filters resulting in higher level of accuracy. Though the level of accuracy was improved the threshold value had to be set for all digital fundus images.

A model that used 2D Gabor wavelet for enhancement of vessels [5] was presented for significant segmentation of vessel. With this, the proposed segmentation model proved to extract the thinnest vessels even for large differentiation in illumination. Though accuracy was obtained, the time with which the accuracy was performed increased with the segmentation of vessel.

Diabetic retinopathy which is considered as the critical eye can significantly minimize the danger of blindness in these patients by 50%. Optimally Adjusted Morphological Operator (OAMO) [6] investigated the states of the patients and presented Optimally Adjusted Morphological Operators for efficient detection of exudates on diabetic retinopathy patients. One of the main drawbacks of this method was that these mechanically detected exudates did not include detailed feature selection technique for diabetic retinopathy detection.

Effective diagnosis and measures for treating diabetic retinopathy (DR) in young adults have improved a lot in the recent years. In [7], summary of methods was provided for the early detection and treatment of DR. A framework [8] was designed for designing benchmark databases and novel measures for the protocol design for treatment of diabetic retinopathy in medical image analysis.

In [9], an image processing technique using adaptive histogram equalization was presented for efficient detection of the presence of exudates in digital fundus images. Followed by this, clustering algorithms were applied for significant segmentation of the exudates and were provided as inputs to Echo State Neural Network (ESNN) to provide an efficient differentiation between the normal and affected digital fundus images. Though the results proved to classify the lesion in accurate manner, various symptoms of diabetic retinopathy were not detected. Detection of microaneurysms (MAs) was used as a key for early detection of diabetic retinopathy.

In [10], a threefold mechanism was presented for early MA detection with the help of filter banks resulting in higher accuracy. In [11], measures were taken to investigate a group of morphological operators used for early detection of microaneurysm on the nondilated pupil in addition to the low-contrast retinal images resulting in precision and accuracy. Nonproliferative Diabetic Retinopathy (NPDR) [12] identified features in exudates from digital fundus image using segmentation based on the extraction of features and classification was performed on the basis of intensity level of the feature and exudates frequency measures. Results also confirmed the level of sensitivity and specificity reducing the diabetic retinopathy.

Based on the aforementioned techniques and methods, an efficient mechanism of diagnosing and ranking the disease level of diabetic retinopathy using Diabetic Fundus Image Recuperation (DFIR) method based on sliding window approach is presented. The contributions of DFIR method include the following:

1. to efficiently diagnose the diseased fundus images using DFIR method and observe the level of severity using the ranking model based on sliding window approach;
2. to diagnose the diabetic retinopathy disease state during the initial phase by selecting the features of optic cup in digital retinal fundus images;
3. to provide more detailed information of features using group sparsity nonoverlapping function with varying range based on the histogram value;
4. to effectively rank the diabetic retinopathy disease level using support vector model based on Spiral Basis Function;
5. to provide a promising result for developing practically automated and assisted diabetic retinopathy diagnosis system.

The organization of the paper is as follows.  Section 2 provides the framework for diagnosing the disease level using image recuperation approach followed by a detailed algorithm and architecture diagram.  Section 3 provides the experimental setups, the result analysis followed by the detailed comparisons with other state-of-the-art methods.  Section 4 includes the related works and finally, Section 5 includes the concluding remarks.

## 2. Materials and Methods

### 2.1. Diabetic Fundus Image Recuperation Approach for Diagnosing Disease Level

In this section, an efficient method for diagnosing disease level for diabetic fundus image using recuperation approach is explained with the help of a neat architecture diagram. The work starts with the designing of sliding window using DFIR method, followed by the identification of disease using optic cup, and finally the designing of a support vector model for disease ranking.

Exudates are one of the most ordinary occurring lesions in diabetic retinopathy fundus images. The shape, intensity, and position of exudates differ a lot amongst diverse diabetic retinopathy patients. The existing diabetic macular edema included information related to blood vessels, length, width, tortuosity, and branching pattern but it did not provide much information related to pathological feature changes and also did not consider the disease severity. The DFIR method on the other hand analyzed the disease severity using the Spiral Basis Function. Initially, sliding window in DFIR method divides the digital fundus images into sliding window blocks of varying dimensional length. The size of digital fundus images varies according to the different set of patients and accordingly the dimensional length also varies.

**Figure 1:**
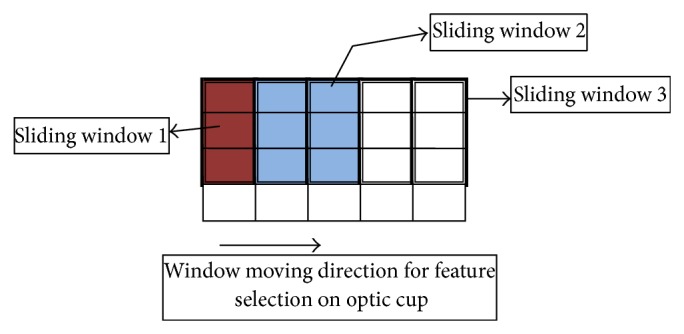
Sliding window on DFIR method.

Figure 1 clearly describes the DFIR method that is divided into blocks based on sliding window processing. The sliding window based feature selection in DFIR method initially divides the optic cup digital fundus images into blocks of specific window size. The overall sliding window blocks evaluate the two sets of histogram results using the DFIR method. The first set of histogram values works from the left boundary of block whereas the other set works from the right boundary of the block in the fundus images. The result obtained from the two sets is combined into one operational set using the Group Sparsity Nonoverlapping Function in DFIR method.

The DFIR method uses the Group Sparsity Function with nonoverlapping pixel for evaluating the histogram intensity range. With the aid of histogram intensity usage, the features are easily spotted on the digital fundus images. Group Sparsity Nonoverlapping Function removes the overlapping of pixel values, and as a result the feature is selected with the minimal time period.

The selected features are used in the second phase of the DFIR method to analyze the disease severity using the ranking level. To measure the ranking level, support vector model is used in DFIR method. The Spiral Basis Function identifies the distance of the disease affected area in the digital fundus image and ranks the patients severity level.

**Figure 2:**
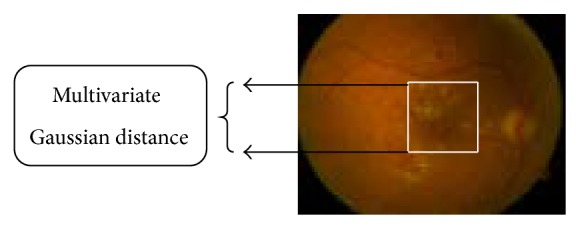
Distance computation on affected diseased optic cup.

**Figure 3:**
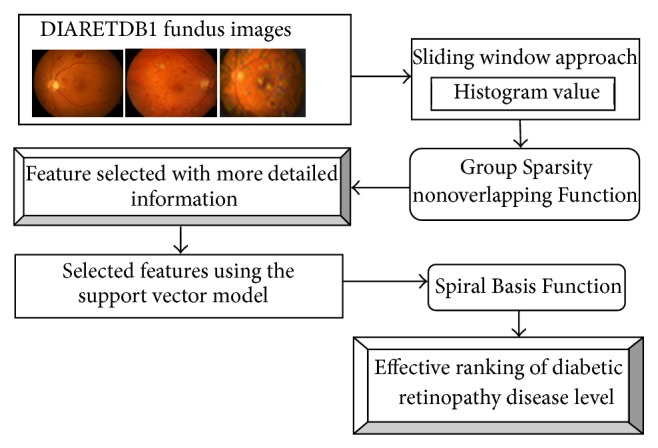
Architecture diagram of DFIR method.

Figure 2 clearly described the distance, that is, severity level on digital fundus images. Spiral Basis Function uses the multivariate Gaussian distance for identifying the depth of affected level on the digital images. The multivariate Gaussian distance clearly describes the level of disease and rank based on the computation value. The overall architecture diagram of Diabetic Fundus Image Recuperation (DFIR) method is depicted in Figure 3.

Figure 3 clearly describes the DFIR method for diagnosing the diseased fundus images and examines the level of severity using the ranking model. Initially, the DIARETDB1 fundus images database is taken for the experimental work. The images use the sliding window approach to divide the fundus images into blocks and then the two sets of histogram values are computed. The computed histogram value uses the Group Sparsity Nonoverlapping Function to remove the overlapping of pixels on computation. The Group Sparsity Nonoverlapping Function chooses the features and offers the detailed feature information. The detailed information uses the support vector model to rank the diabetic retinopathy disease level. Finally, Spiral Basis Function is used to compute the distance, that is, depth of affected area using the multivariate Gaussian distance.

### 2.2. Sliding Window Approach

Sliding window approach is used in localizing the features from dividing the fundus images into blocks {*b*_1_, *b*_2_, and *b*_3_ ⋯ *b*_*n*_} of size “*n*.” The rectangular shaped boundary is selected for sliding window operation in DFIR method and rectangular shape division operation is carried out in fundus images. The window of “*n*” size contains the block such that

(1) $$\begin{array}{c} b_{1}=\text{op}\left(a_{11},a_{12}\right)\\ b_{2}=\text{op}\left(a_{12},a_{13}\right)\\ \vdots \\ b_{n}=\text{op}\left(a_{13},a_{1n}\right). \end{array}$$

Equation (1) describes the sliding window division operation on the optic cup “op” into “*n*” blocks. In DFIR method, {*a*_11_, *a*_12_, *a*_13_, …,*a*_1*n*_}  is the divided boundary on “*n*” dimensional digital fundus mages. The histogram intensity value range of the left and the right boundary is computed as

(2) $$\begin{array}{c} \text{LB}\left(\text{Histogram}\right)=\frac{{\text{FI}}_{\text{cols}}{\text{FI}}_{\text{rows}}}{S_{\max}+1},\\ \text{RB}\left(\text{Histogram}\right)=\frac{{\text{FI}}_{\text{rows}}{\text{FI}}_{\text{cols}}}{S_{\max}-1}. \end{array}$$

The “LB” and “RB” are the left and right boundary of the fundus images to compute the histogram intensity value. The intensity range is measured based on rows and column pixel of the digital image. *S*_max⁡_ in DFIR method is the maximum group sparsity value used for the histogram value computation. In the left boundary range, the value is incremented as the window moves from the left end to the right end of the image. In the right boundary range, the value is decremented as the window moves from the right end to the left end of the image. The algorithmic step of the sliding window operation is described as follows: 

//Sliding Window

**Begin**

*Input*. Digital Fundus Images from DIARETDB1 database

*Output*. Feature Selected with detailed optic cup information

*Step 1*. Divided into block of size “*n*” such that *b*_1_, *b*_2_, and *b*_3_ ⋯ *b*_*n*_

*Step 2*. Compute *b*_*n*_ = op(*a*_13_,…, *a*_1*n*_)

*Step 3*. For *i* = Row point 1 to *n*

*Step 4*. For *j* = column point 1 to *n*

*Step 5*. Histogram intensity value of left boundary form is LB(Histogram) = FI_cols_FI_rows_/(*S*_max⁡_ + 1)

*Step 6*. Histogram intensity value of right boundary form is RB(Histogram) = FI_rows_FI_cols_/(*S*_max⁡_ − 1)

*Step 7*. Compute Group Sparsity function to avoid overlapping of pixels

*Step 8*. Window Fundus image = LB(Histogram) + RB(Histogram).

*Step 9*. End For *j*

*Step 10*. End For *i*

**End**

The above algorithm clearly describes the sliding window through the algorithmic step with for loop structure. The histogram intensity value of left and right boundary is computed in DFIR method. The two sets of values are combined to remove the overlapping condition in Step 8 using the Group Sparsity Nonoverlapping Function. Group Sparsity Nonoverlapping Function is clearly described in Section 2.2.1.

#### 2.2.1. Group Sparsity Nonoverlapping Function

The optic cup histogram value from the right and left boundary is combined together to reduce the feature selection time and Group Sparsity Nonoverlapping Function is computed as

(3) Group  Sparsity  Nonoverlapping  Function  (GS) =∑i=1nS1,2,…,max⁡2.

“*S*” denotes the sparsity histogram value from window 1,2,…, max⁡ value. The group Sparsity Nonoverlapping Function (GS) is squared where the left and right boundary values are combined together. The feature selection time taken using DFIR method is formularized as

(4) $$\begin{array}{c} \text{Feature}\;\text{Selection}\;\text{Time}=\overset{n}{\underset{i,j=1}{\sum }}\left\|f_{i}^{T}\text{LB}\left(\text{FI}\right)+\text{RB}\left(\text{FI}\right)\right\|. \end{array}$$

The combined histogram value on time “*T*” produces the result with features *f*_*i*_. LB(FI) + RB(FI) is a combined value without any overlapping of pixel value.

### 2.3. Support Vector Model

The DFIR machine learning approach uses the support vector model to rank the disease severity level from the digital fundus images. The feature selected information is used in the support vector model to identify the position of the disease affected area in fundus images. Spiral Basis Function is used for the ranking of diseased fundus images using the DFIR method.

#### 2.3.1. Spiral Basis Function

The Spiral Basis Function in support vector model of DFIR method uses the multivariate Gaussian distance to identify the depth of the disease affected position. The depth level points out the ranking of retinopathy disease level. The Spiral Basis Function with multivariate Gaussian distance is denoted as

(5) Multivariate  Gaussian  distance =1−2σ1σ2σ12+σ22e−1/4μ1−μ22σ12+σ22.

The Gaussian distance with *σ*_1_ and *σ*_2_ denotes the depth of the affected portion in the fundus image from left to right position. *μ*_1_ and *μ*_2_ denote the length value to which the disease gets spread on the four directions denoted by 1/4. The distance is computed and threshold value is set to rank the disease level. If the computed (5) value is higher than the threshold value, then the rank gets increased; that is, the disease severity level gets increased. The ranking in DFIR method develops a practical assisted diabetic retinopathy disease ranking system.

## 3. Experimental Evaluation

DFIR method is developed in MATLAB platform. DFIR method uses the DIARETDB1-Standard Diabetic Retinopathy Database. DIARETDB1 database is a public database used for benchmarking diabetic retinopathy detection. The idea of the DFIR method is to define a database with a test image used as benchmark images for feature selection. The DIARETDB1 database includes 89 color fundus images. Out of 89 color fundus images, 84 contain at least mild nonproliferative signs of the diabetic retinopathy. The remaining 5 color fundus images are considered as normal which do not contain any signs of diabetic retinopathy. The method DFIR is evaluated using 35 color fundus images collected from 40 different patients.

### 3.1. Experimental Analysis of DFIR Method

By using DIARETDB1 database, the defined testing method results are compared with existing method. DFIR method is compared in the existing Feature Based Macular Edema Detection (FMED) [1] and Optimally Adjusted Morphological Operator (OAMO) [2]. The experiment is conducted on factors such as sensitivity, specificity rate, ranking efficiency, and feature selection time. The sensitivity of DFIR method measures in (6) the capability of diagnosing those patients with the retinopathy disease (in terms of %), also referred to as a measure for providing a positive test with retinopathy disease:

(6) $$\begin{array}{c} \text{Sensitivity}=\frac{\text{True}\;\text{Positive}}{\text{True}\;\text{Positives}+\text{False}\;\text{Negatives}}, \end{array}$$

where True  Positive refers to the number of diabetic retinopathy diseases; False  Negatives refer to the number of diabetic retinopathy diseases that were not detected. The specificity of DFIR method measures the capability to correctly identify those patients without retinopathy disease (in terms of %), also referred to as a measure for providing a negative test with retinopathy disease:

(7) $$\begin{array}{c} \text{Specificity}=\frac{\text{True}\;\text{Negatives}\;}{\text{True}\;\text{Negatives}+\text{False}\;\text{Positives}\;}, \end{array}$$

where True  Negatives refer to the number of nondiabetic retinopathy diseases which were correctly identified as nondiabetic retinopathy whereas False  Positives measure the number of nondiabetic retinopathy diseases which are detected wrongly as diabetic retinopathy disease. Ranking efficiency using DFIR method is measured using the support vector model based on Spiral Basis Function evaluated using (3). The ranking efficiency is measured in terms of percentage (%). The time taken to select the feature using DFIR method is evaluated from (4) which is measured in terms of milliseconds (ms).

### 3.2. Result Analysis of BFIR

The result analysis of BFIR for Diabetic Fundus Image Recuperation (DFIR) method using Standard Diabetic Retinopathy Database is compared with existing Feature Based Macular Edema Detection (FMED) [2] and Optimally Adjusted Morphological Operator (OAMO) [6].  Table 1 represents the sensitivity rate obtained using MATLAB simulator and comparison is made with two other methods, namely, FMED [2] and OAMO [6].

**Figure 4:**
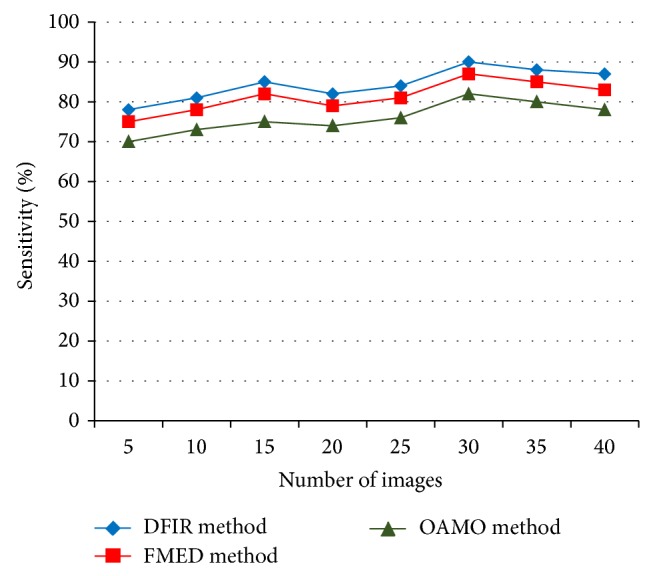
Measure of sensitivity.

Figure 4 shows that the proposed DFIR method provides higher sensitivity rate when compared to FMED [2] method and OAMO [6] method. This is because of the application of sliding windows in DFIR method with varying range that obtains the features based on the histogram value that provides more elaborated information of features using Group Sparsity Nonoverlapping Function improving the sensitivity value by 2–4% when compared to FMED. In addition to that, the use of intensity range based on row and column pixel of the digital image with left and right boundaries increases the rate of Sensitivity by 8–11% when compared to the OAMO [6] method.

The comparison of specificity is presented in Table 2 with respect to the number of images in the range of 5–40. With increase in the number of images, the specificity also gets increased.

**Figure 5:**
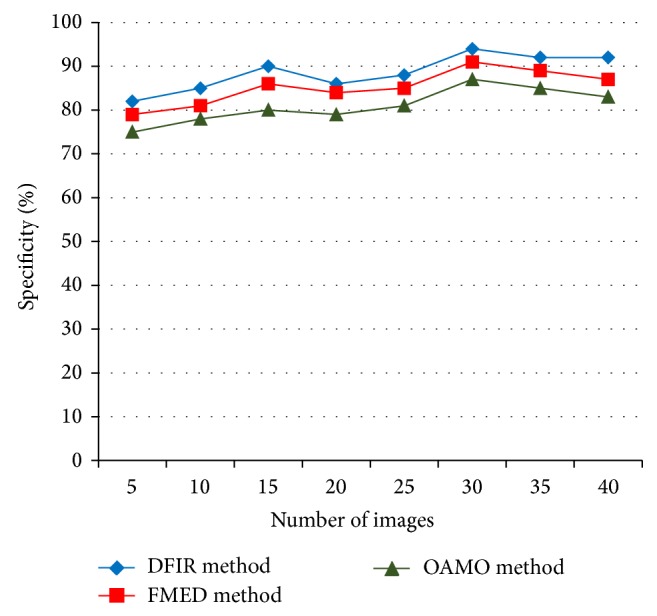
Measure of specificity.

To ascertain the performance of the specificity, comparison is made with two other existing methods, Feature Based Macular Edema Detection (FMED) [2] and Optimally Adjusted Morphological Operator (OAMO) [6]. In Figure 5, the number of images is varied between 5 and 40. From the figure it is illustrated that the specificity is higher or increased using the proposed DFIR method when compared to the two other existing methods. This is because with the application of support vector model the DFIR method based on Spiral Basis Function effectively ranks the diabetic retinopathy disease level, therefore increasing the specificity by 3–5% when compared to FMED [2] method. Furthermore, by ranking the disease level on each candidate set, with the help of Spiral Basis Function, the specificity is increased considerably using DFIR method by 7–11% than when compared to OAMO [6] method.

The ranking efficiency for DFIR method is elaborated in Table 3. We consider the method with sliding windows of size 7 for experimental purpose using MATLAB.

**Figure 6:**
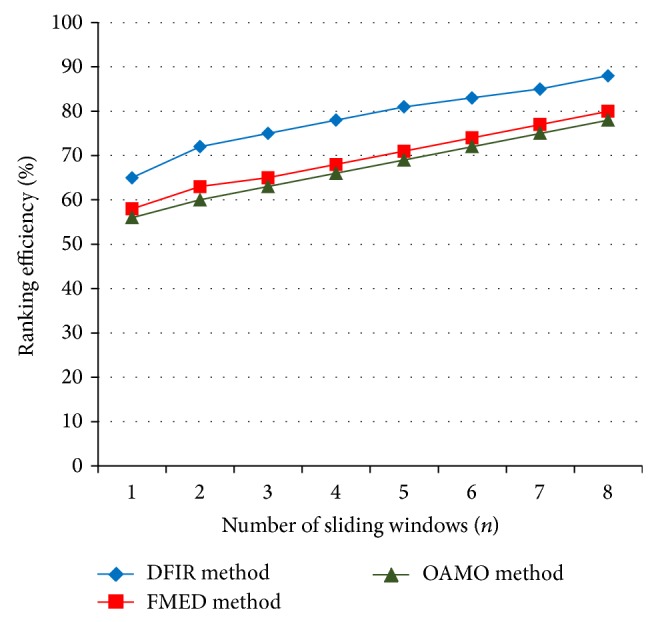
Measure of ranking efficiency.

In Figure 6, we depict the ranking efficiency attained using the sliding windows of size 1 to 8 for experimental purposes. From the figure, the value of ranking achieved using the proposed DFIR method is higher when compared to the other two existing methods, Feature Based Macular Edema Detection (FMED) [2] and Optimally Adjusted Morphological Operator (OAMO) [6]. Besides we can also observe that, by increasing the size of the sliding windows, the ranking efficiency value is increased using all the methods. But comparatively it is higher in DFIR method because, the depth of the disease affected area are efficiently identified using the Spiral Basis function which intern uses the Multivariate Gaussian distance. With the identified depth level points, the ranking of retinopathy disease level is improved by 9–13% compared to FMED [2]. Furthermore, use of Spiral Basis function ranks the patients disease severity level and improves it by 11–16% than compared to the OAMO [6] method.

**Figure 7:**
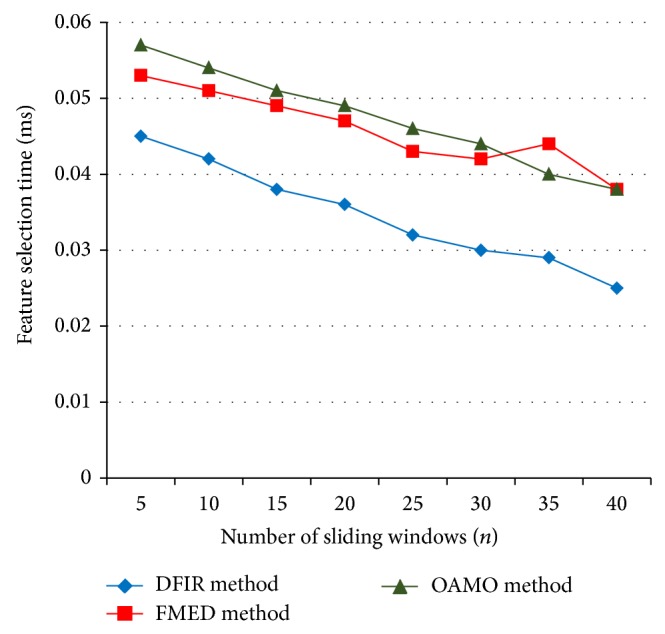
Measure of feature selection time.

Table 4 and Figure 7 illustrate the feature selection time versus the number of sliding windows measured in terms of milliseconds for experimental purpose conducted using MATLAB. From the figure we can note that the feature selection time attains 34.375% improvement for the sliding window of size 25 when compared to FMED [2] method and 43.75% improvement when compared to OAMO [6] method which shows that there is a significant gain using the proposed DFIR method. This is because the Group Sparsity Function with nonoverlapping pixel evaluates the histogram intensity range and with the aid of histogram intensity the feature is selected with minimal time period. Further, the two sets of values, that is, the left and right boundary, are combined to remove the overlapping condition by minimizing the feature selection time by 17–50% when compared to FMED [2] method and 26–52% compared to the OAMO [6] method.

## 4. Related Works

The major breakthrough of blindness is caused due to the diabetic retinopathy. Early identification and proper meditation can prevent the disease nearing the individual and avoid blindness to certain extent. One of the dangerous retinal injuries, hazardous by gazing of sun, is called the solar retinopathy which occurs with the observation of solar eclipse observation without any precautious measure. At the same time, one of the safest ways to view the solar eclipse is with the indirect projection of image as suggested by many researchers.

Fourier-domain optical coherence tomography (OCT) [13] was introduced as the imaging tool to prevent retinal damage by the individuals. Though proved to be an efficient tool, it was opted only for the left eye. A hybrid approach for both left and right eyes called the combined cross point number [14] was designed from retina fundus images for efficient diagnosing of diabetic retinopathy. Results when applied to retina fundus images proved to be highly precise and accurate with minimized false error rate for treating diabetic retinopathy. In [15], different measures were taken to identify the concentrations in patients suffering from type 2 diabetes mellitus and diabetic retinopathy and significantly measured the relationship between purine metabolites and disease.

Differential Morphological Profile (DMP) [16] structured an automatic method for efficient detection of exudates from digital color fundus images using three major phases. Gaussian smoothing and contrast enhancement were performed in the initial stage, followed by which DMP was applied on the features being extracted. Finally, actual exudates were obtained on the basis of the location of optic disc, shape index, and area resulting in accuracy. Microperimetry [17] has the added advantage of combining the functional parameter with the morphologic status of retina.

With the application of microperimetry, the changes observed in the level of diabetes were efficiently measured compared to that of DME. But accurate results were not obtained. To obtain accurate results, different techniques including normalization of images, efficient compactness classification model, significant morphology operations, process of filtering using Gaussian process, and threshold factor were introduced for early detection of neovascularization. To improve the accuracy of the results obtained, a region-based neovascularization classification method was applied.

## 5. Conclusion

Diagnosing and ranking retinopathy disease level has become the key for digital fundus images to achieve minimum error rate and improve the level of sensitivity with relatively lesser amount of feature selection time. In this work, we investigate the performance effects of machine learning methods to categorize the candidate exudates fundus images and propose a method called Diabetic Fundus Image Recuperation (DFIR) based on sliding window approach in digital retinal fundus images that minimizes the effects of sensitivity and greatly improves the ranking efficiency. First, we study the use of sliding window blocks to divide fundus images into blocks and propose using the rectangular shape division operation as the window moving direction for feature selection in DFIR. Second, we develop several Group Sparsity Functions with nonoverlapping pixel for evaluating the histogram intensity range that work with the feature selection module to minimize the feature selection time. We also integrate Spiral Basis Function with the features selected using support vector model to effectively rank the diabetic retinopathy disease using DIARETDB1-Standard Diabetic Retinopathy Database. The experiment conducted using DIARETDB1 shows that the DFIR method achieves up to 35 percent improvement on sensitivity compared to the state-of-the-art methods.

## Figures and Tables

**Table 1:** Tabulation for sensitivity.

Number of images	Sensitivity (%)		
	DFIR method	FMED method	OAMO method
5	78	75	70
10	81	78	73
15	85	82	75
20	82	79	74
25	84	81	76
30	90	87	82
35	88	85	80
40	87	83	78

**Table 2:** Tabulation for specificity.

Number of images	Specificity (%)		
	DFIR method	FMED method	OAMO method
5	82	79	75
10	85	81	78
15	90	86	80
20	86	84	79
25	88	85	81
30	94	91	87
35	92	89	85
40	92	87	83

**Table 3:** Tabulation for ranking efficiency.

Number of sliding windows (*n*)	Ranking efficiency (%)		
	DFIR method	FMED method	OAMO method
1	65	58	56
2	72	63	60
3	75	65	63
4	78	68	66
5	81	71	69
6	83	74	72
7	85	77	75
8	88	80	78

**Table 4:** Tabulation for feature selection time.

Number of sliding windows (*n*)	Feature selection time (ms)		
	DFIR method	FMED method	OAMO method
5	0.045	0.053	0.057
10	0.042	0.051	0.054
15	0.038	0.049	0.051
20	0.036	0.047	0.049
25	0.032	0.043	0.046
30	0.030	0.042	0.044
35	0.029	0.044	0.040
40	0.025	0.038	0.038
